# Rapidly Progressive Interstitial Lung Disease With Pneumothorax and Pneumomediastinum Secondary to Amyopathic Dermatomyositis

**DOI:** 10.7759/cureus.50780

**Published:** 2023-12-19

**Authors:** Aboubekr Imzil, Naoufal Assoufi, Souhail Mouline, Abdenasser El Kharras, Hicham Naji-Amrani

**Affiliations:** 1 Pneumo-Phtisiology Department, Souss-Massa University Hospital Center, Medical and Pharmacological Faculty, Ibn Zohr University, Agadir, MAR; 2 Internal Medicine Department, Oued Eddahab Military Hospital, Medical and Pharmacological Faculty, Mohammed V University, Rabat, MAR; 3 Laboratory Medicine Department, Oued Eddahab Military Hospital, Medical and Pharmacological Faculty, Cadi Ayyad University, Marrakech, MAR; 4 Radiology Department, Oued Eddahab Military Hospital, Medical and Pharmacological Faculty, Mohammed V University, Rabat, MAR; 5 Pneumo-Phtisiology Department, Oued Eddahab Military Hospital, Medical and Pharmacological Faculty, Sidi Mohammed Benabdellah University, Fes, MAR

**Keywords:** prognosis, anti-mda-5 antibodies, interstitial lung disease, cutaneous lesions, amyopathic dermatomyositis

## Abstract

Amyopathic dermatomyositis is a rare form of dermatomyositis characterized by cutaneous lesions without clinical, biological, or histological muscular involvement. Pulmonary complications associated with this condition are diffuse interstitial lung disease (ILD), pneumomediastinum, and spontaneous pneumothorax. The form associated with anti-melanoma differentiation-associated protein 5 (anti-MDA-5) antibodies is reputed to have a poor prognosis and is responsible for ILD which can rapidly progress to fatal respiratory failure. Treatment of amyopathic dermatomyositis is essentially based on corticosteroid therapy and immunosuppressants. We present the case of a 42-year-old patient followed for three months for diffuse ILD. The patient was hospitalized for respiratory distress secondary to an aggravation of ILD with pneumomediastinum and pneumothorax. The patient also presented with cutaneous lesions identified as Gottron’s papules. The patient was diagnosed with amyopathic dermatomyositis positive for anti-MDA-5 antibodies.

## Introduction

Dermatomyositis is a rare connective tissue disease of unknown cause, defined by an idiopathic inflammatory disease of the muscles associated with cutaneous manifestations. This connective tissue disease can be complicated at the respiratory level by interstitial lung disease (ILD), which is a frequent complication [1]. Other complications reported less frequently include pneumomediastinum and spontaneous pneumothorax [2]. The clinically amyopathic form of dermatomyositis, described in the 1990s [3], is considered to have a poor prognosis and is associated with ILD which is frequently inaugural, rapidly progressive, less responsive to treatment, and associated with increased mortality [4,5]. In this article, we report a case of amyopathic dermatomyositis (ADM) complicated by ILD with pneumothorax and pneumomediastinum.

## Case presentation

**Figure 1:**
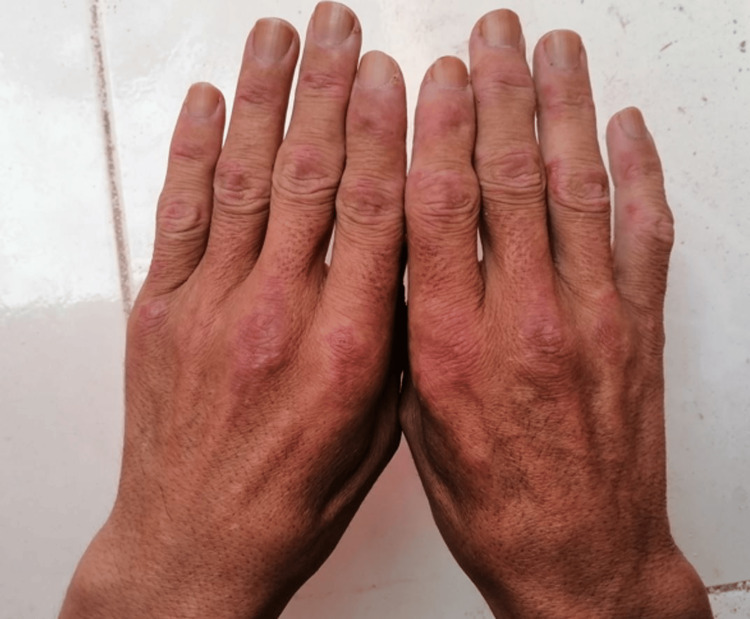
Gottron’s papules: erythematous lesions on the extensor surfaces of proximal interphalangeal and metacarpophalangeal joints.

A 42-year-old man, employed as a sailor, followed for diffuse ILD for three months. ILD evolution was marked by a progressive aggravation of dyspnea (Sadoul stage 4) and subcutaneous swelling on the neck and trunk without muscular weakness, xerostomia, xerophthalmia, or fever. A physical examination revealed a respiratory rate of 25 cycles/minute and oxygen saturation at 90% increasing to 95% on 5 L. Additionally, he had bilateral crepitus rales; subcutaneous emphysema in the trunk, neck, and submandibular region; and erythematous lesions on the extensor surfaces of the interphalangeal and proximal metacarpophalangeal joints (Figure 1).

**Figure 2:**
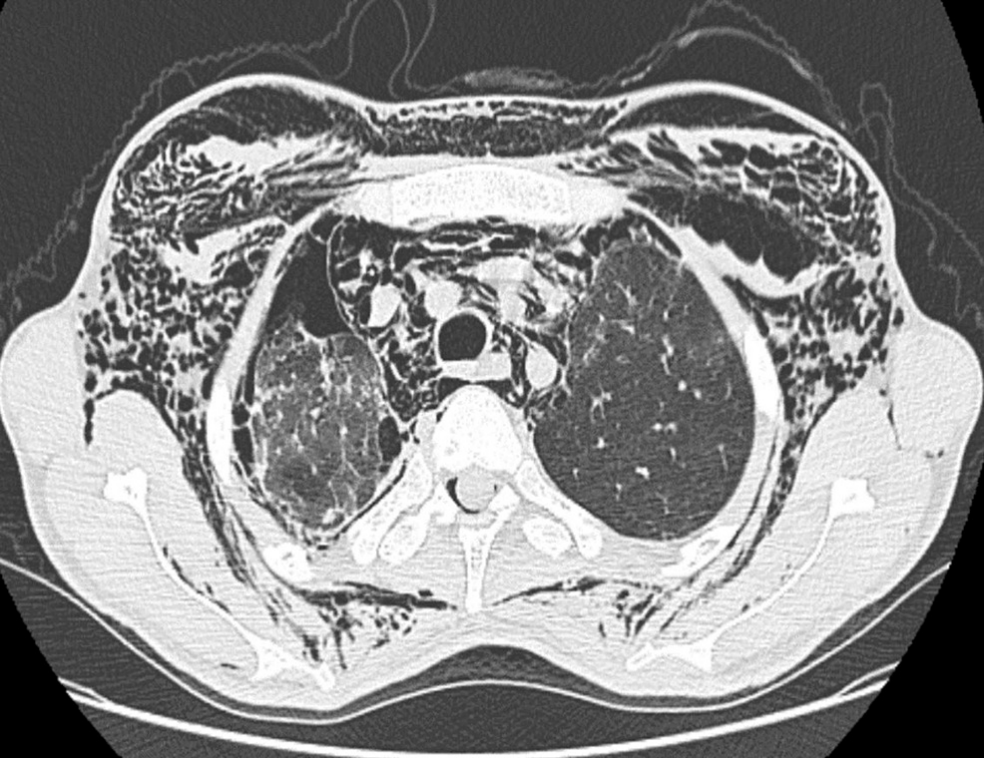
Chest scan (axial sections through the parenchymal window at the cervical region) showing subcutaneous emphysema, right pneumothorax, pneumomediastinum, and pneumorrhachis associated with diffuse interstitial lung disease.

**Figure 3:**
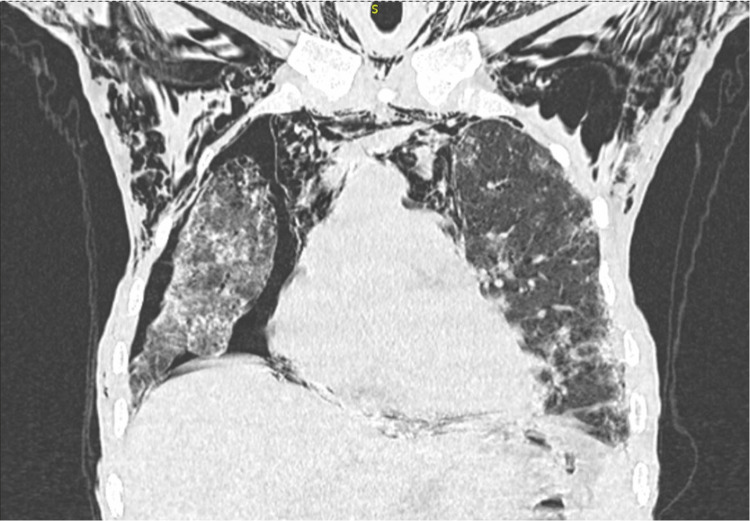
Chest scan (coronal reconstruction) showing subcutaneous emphysema, right pneumothorax, and pneumomediastinum with diffuse interstitial lung disease.

**Figure 4:**
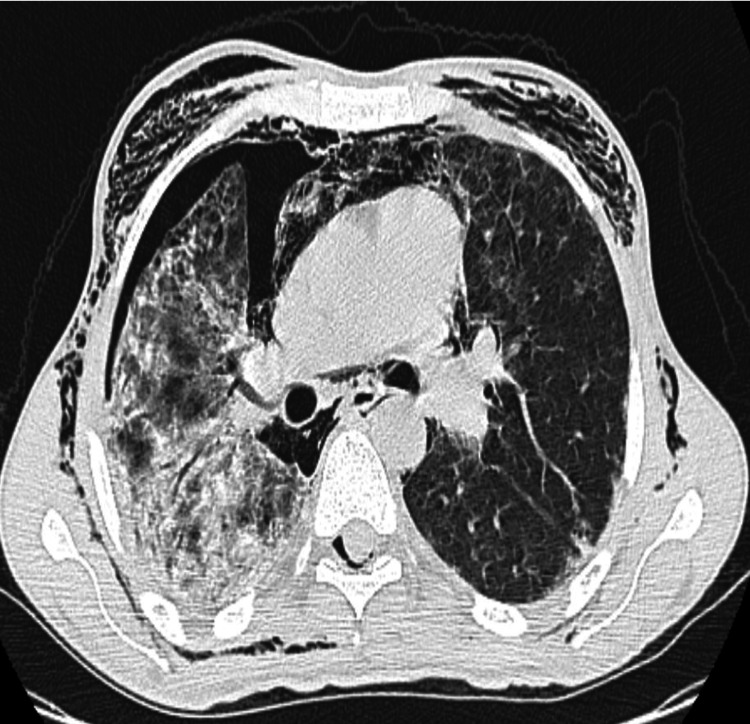
Chest scan (axial through the parenchymal window) showing foci of alveolar condensation associated with ground-glass areas and septal thickening, more marked on the right.

A frontal chest X-ray showed a minor right pneumothorax, bilateral alveolar-interstitial syndrome, and subcutaneous emphysema. A thoracic scan with helical acquisition over the chest was performed, which revealed a right pneumothorax of moderate size; a pneumomediastinum of moderate size; multiple foci of alveolar condensation, peripheral and bilateral, associated with ground-glass areas and septal thickening more marked on the right; and subcutaneous emphysema in the cervical and thoracic soft tissue with local fusing at the spinal canal (Figures 2-4).

The biological tests showed a normal white blood cell count (6,000 mm^3^), lymphopenia (200 mm^3^), normal creatine phosphokinase (83 U/L), elevated C-reactive protein (107 mg/L), negative HIV serology, normal fasting blood glucose, positive MDA-1 (17 U/mL), and positive anti-Sjögren's-syndrome-related antigen A autoantibodies (83 U/mL). All other biological test results were normal. An electroneuromyogram also showed normal results, and the anatomopathological study of the muscle tissue showed no histological abnormalities.

A chest drain was placed, with the lung returning to the chest wall and a reduction in subcutaneous emphysema. The patient was put on oxygen therapy, antibiotic therapy, injectable methylprednisolone, and analgesic treatment. With no clinical or radiological improvement, the patient received three boluses of methylprednisolone (1,000 g) per day for three successive days. A bolus of cyclophosphamide was planned, but the patient developed respiratory distress, after which he died.

## Discussion

ADM is rare, representing around 25% of dermatomyositis cases. It affects women more often than men. It mainly affects young adults, and juvenile-onset cases have been reported in the literature [6]. ADM is characterized by cutaneous lesions highly suggestive of dermatomyositis without clinical, biological, or histological muscle involvement (normal electromyogram, muscle enzymes, and muscle biopsy) [7]. Heliotropic erythema and Gottron’s papules are the most frequently observed cutaneous manifestations, seen in about 80% of cases [6]. Patients with ADM have a higher risk of developing ILD, which can rapidly progress to fatal respiratory failure [5]. In the absence of clinical, biological, histological, and electromyographic muscle abnormalities and the presence of ILD with Gottron’s papules, we concluded that our patient had ADM.

Recently, a new phenotype of ADM has been identified as being related to the identification of anti-melanoma differentiation-associated protein 5 (anti-MDA-5) antibodies [8]. This antibody is a non-invasive biomarker for the diagnosis of rapidly progressive ILD in patients with dermatomyositis [9]. The anti-MDA-5 antibody was positive in our patient, and it was noted that he presented with rapid aggravation of dyspnea over a three-month period.

Pneumomediastinum occurs in 11.8% of dermatomyositis cases. It appears with a median delay of 8 months (3-26 months) and is accompanied by pneumothorax in 40% of cases. An increased prevalence of pneumomediastinum in amyopathic forms of dermatomyositis has been observed, particularly in the presence of anti-MDA-5 antibodies [10]. Our patient also had both pneumothorax and pneumomediastinum.

The rupture of subpleural blebs and cysts that develop from interstitial fibrosis and a raised intra-alveolar pressure have been speculated as potential risk factors for developing pneumomediastinum. Corticosteroid treatment also may lead to pneumomediastinum by weakening alveolar walls [11].

The prognosis for ADM with anti-MDA-5 autoantibodies is poor, especially if the ILD is rapidly progressive. Mortality is high, with 20% to 30% of patients dying within the first six months of the disease, most from pulmonary complications. ILD appears to stop progressing in patients who survive beyond six months. In a recent series, very few patients with ADM with anti-MDA-5 autoantibodies had an associated cancer. Our patient died following an aggravation of respiratory disease; a neoplastic process was not identified in our patient [12].

The treatment of ADM has not yet been standardized and is currently based on high-dose corticosteroid therapy (1 mg/kg of prednisone) combined with immunosuppressants or used as a second-line treatment. Some centers use a triple-therapy combination (corticosteroids, cyclophosphamide, and calcineurin inhibitors such as cyclosporine A or tacrolimus) from the start of the disease. Interestingly, a recent study has suggested that rituximab may be a useful treatment for patients with ADM related to rapidly progressive ILD with anti-MDA-5; however, pulmonary infection is a major risk [13].

## Conclusions

ADM is a rare pathology that is frequently diagnosed late. The association of rapidly progressive ILD with cutaneous lesions, in particular heliotropic erythema and Gottron’s papules, along with minor muscle expression and the positivity of anti-MDA-5 autoantibodies should lead to suspicion of this pathology. Early treatment may improve the prognosis of the disease.

## References

[1] Callen JP (2000). Dermatomyositis. Lancet.

[2] Marie I, Dominique S (2006). [Pulmonary damage during polymyositis and dermatomyositis: interstitial lung disease]. Presse Med.

[3] Euwer RL, Sontheimer RD (1991). Amyopathic dermatomyositis (dermatomyositis siné myositis). Presentation of six new cases and review of the literature. J Am Acad Dermatol.

[4] Suda T, Fujisawa T, Enomoto N (2006). Interstitial lung diseases associated with amyopathic dermatomyositis. Eur Respir J.

[5] Mukae H, Ishimoto H, Sakamoto N (2009). Clinical differences between interstitial lung disease associated with clinically amyopathic dermatomyositis and classic dermatomyositis. Chest.

[6] Caproni M, Cardinali C, Parodi A (2002). Amyopathic dermatomyositis: a review by the Italian Group of Immunodermatology. Arch Dermatol.

[7] Sontheimer RD (1999). Cutaneous features of classic dermatomyositis and amyopathic dermatomyositis. Curr Opin Rheumatol.

[8] Ceribelli A, Fredi M, Taraborelli M (2014). Prevalence and clinical significance of anti-MDA5 antibodies in European patients with polymyositis/dermatomyositis. Clin Exp Rheumatol.

[9] Li L, Wang Q, Wen X (2017). Assessment of anti-MDA5 antibody as a diagnostic biomarker in patients with dermatomyositis-associated interstitial lung disease or rapidly progressive interstitial lung disease. Oncotarget.

[10] Ma X, Chen Z, Hu W, Guo Z, Wang Y, Kuwana M, Sun L (2016). Clinical and serological features of patients with dermatomyositis complicated by spontaneous pneumomediastinum. Clin Rheumatol.

[11] Neves Fde S, Shinjo SK, Carvalho JF, Levy-Neto M, Borges CT (2007). Spontaneous pneumomediastinum and dermatomyositis may be a not so rare association: report of a case and review of the literature. Clin Rheumatol.

[12] Uzunhan Y, Nunes H, Leroux G, Miyara M, Benveniste O, Allenbach Y (2016). Dermato-pulmonary syndrome associated with MDA-5 antibodies. Eur Respir J.

[13] Kohsaka H, Mimori T, Kanda T (2019). Treatment consensus for management of polymyositis and dermatomyositis among rheumatologists, neurologists and dermatologists. J Dermatol.

